# The Cost of Out-Patients at Special Hospitals

**Published:** 1892-09-03

**Authors:** 


### Editor's Letter Box.

[Our correspondents are reminded that prolixity of statement is a great bar to publication, and that brevity of style and conciseness of statement greatly facilitate early insertion.]

### THE COST OF OUT-PATIENTS AT SPECIAL HOSPITALS.

Mr. Lennox Browne writes: On examination of the statistics of the Hospital Sunday Fund, published for the use of the Council, it appears that while the expense of the out-patients at the 55 special hospitals detailed therein averages 3s. 10¾d. per head, the cost of each out-patient at the Central London Throat and Ear Hospital is printed in the same list as 3s. 6½d. Taking the 54 dispensaries to whom a grant is made the figures work out to a cost per head of 3s. 5¾d. In the light of these figures it cannot be maintained that there is any justification for the charge of extravagance imputed to this hospital (*vide* editorial note in issue of August 6th, page 320). This charge should, therefore, be frankly withdrawn, and credit given for the economy exercised at this particular hospital.

